# Barriers for pregnant women living in rural, agricultural villages to accessing antenatal care in Cambodia: A community-based cross-sectional study combined with a geographic information system

**DOI:** 10.1371/journal.pone.0194103

**Published:** 2018-03-19

**Authors:** Junko Yasuoka, Keiko Nanishi, Kimiyo Kikuchi, Sumihiro Suzuki, Po Ly, Boukheng Thavrin, Tsutomu Omatsu, Tetsuya Mizutani

**Affiliations:** 1 Research and Education Center for Prevention of Global Infectious Diseases of Animals, Tokyo University of Agriculture and Technology, Tokyo, Japan; 2 Office of International Academic Affairs, Graduate School of Medicine and Faculty of Medicine, The University of Tokyo, Tokyo, Japan; 3 Graduate Education and Research Training Program in Decision Science for Sustainable Society, Kyushu University, Fukuoka, Japan; 4 University of North Texas Health Science Center, University of North Texas, Denton, Texas, United States of America; 5 National Center for Parasitology, Entomology & Malaria Control (CNM), Ministry of Health, Phnom Penh, Cambodia; Boston University School of Public Health, UNITED STATES

## Abstract

**Background:**

Maternal morbidity and mortality is still a major public health issue in low- and middle-income countries such as Cambodia. Improving access to antenatal care (ANC) services for pregnant women has been widely recognized as one of the most effective means of reducing maternal mortality and morbidity. As such, this study examined the barriers for pregnant women living in rural, agricultural villages to accessing ANC based on data collected in the Ratanakiri province, one of the least developed provinces in Cambodia, using a combination of a community-based cross-sectional survey and a geographic information system (GIS).

**Methods:**

A community-based cross-sectional survey was conducted among 377 mothers with children under the age of two living in 62 villages in the Ratanakiri province, Cambodia, in December 2015. Face-to-face interviews were conducted to ask mothers about their ANC service use, knowledge of ANC, barriers to accessing health facilities, and complications they experienced during the most recent pregnancy. At the same time, GIS data were also collected using a Global Positioning System (GPS) to accurately measure actual travel distance of pregnant women to access health facilities and to examine geographical and environmental barriers in greater detail.

**Results:**

Only a third of the mothers met the recommendations made by the World Health Organization (WHO) of receiving ANC four times or more (achieved ANC4+), and a quarter of the mothers had never received ANC during their most recent pregnancy. Factors positively associated with achieving ANC4+ were mother’s secondary or higher education (adjusted odds ratio [AOR] = 5.50, 95% confidence interval [CI]: 1.74, 17.37), being aware that receiving ANC is recommended (AOR = 2.74, 95% CI: 1.25, 6.00), and knowledge about the recommended frequency for ANC (AOR = 2.26, 95% CI: 7.22). Actual travel distance was negatively associated with achieving ANC4+. Mothers who had to travel 10.0–14.9 km were 68% less likely (AOR = 0.32, 95% CI: 0.10, 0.99), and those who had to travel 15.0 km or longer were 79% less likely (AOR = 0.21, 95% CI: 0.07, 0.62) to have achieved ANC 4+, both compared to those who travelled 5.0 km or less. While most previous studies have used a straight-line to measure distance traveled, this study much more accurately measured the actual distance traveled by using a GIS. As a result, there was a statistically significant discrepancy between actual travel distance and straight-line distance.

**Conclusions:**

This study revealed promoting factors and barriers for ANC use among pregnant women living in remote, agricultural villages in Cambodia. Furthermore, this study highlights the importance of measuring travel distances accurately to ensure that targeted interventions for ANC are not misguided by straight-line distances. The methodology used in this study can be applied widely to other developing countries, especially in remote areas with limited road networks where there may be a large discrepancy between actual and straight-line distances.

## Introduction

Maternal morbidity and mortality is still a serious public health issue in low- and middle-income countries, even though there has been remarkable progress in improving maternal health since the Millennium Development Goals (MDGs) were adopted in 2000. An estimated 303,000 women died because of complication of pregnancy and childbirth in 2015, most of which occurred in rural, resource-limited settings in developing countries [1–3]. Lack of access to local health care services, including antenatal care (ANC) services for pregnant women, has been consistently reported as one of the key obstacles to reduce maternal mortality and morbidity [4,5].

The continuum of care (CoC) in maternal, neonatal, and child health (MNCH) has gained much attention as a means to reduce the burden of maternal deaths, neonatal deaths, and deaths among children under five, especially in low- and middle-income countries [6]. Enabling pregnant women to continuously receive ANC, skilled birth attendance at delivery, and postnatal care (PNC) are reported to have a significant positive impact on overall MNCH indicators [7]. In particular, receiving ANC has been shown to be one of, if not the best, indicator of positive pregnancy outcomes, significantly contributing to the reduction of maternal morbidity and mortality [5,8]. ANC are used to identify pregnancy risks and provide care for pregnant women who might be susceptible to a wide variety of potentially fatal conditions such as HIV, malaria, tuberculosis, anemia, and malnutrition [5,9,10]. Additionally, receiving ANC has been found to increase subsequent use of maternal and child health services, especially skilled birth attendance [11,12]. The World Health Organization (WHO) recommends that pregnant women receive ANC at least four times, comprising of tetanus toxoid vaccination, screening of infections, and identification of danger signs of pregnancy complications [13].

However, access to ANC is quite limited in most developing countries, and ANC use is often reported to be low [5]. Variety of barriers to access ANC have been reported in several previous studies [5,8,14–19]: sociocultural and psychological barriers included customs of keeping pregnancy a secret, reliance on traditional/alternative antenatal practices, influence of family members, and reluctance caused by poor attitudes of medical staff [20–25]. ANC use can also be hindered by a lack of knowledge about ANC and benefits of attendance as well as unawareness of health facilities that provide ANC [23,26]. Costs were also reported to be significant barriers: direct costs include medical care fees and transportation fees, while indirect costs include time off work and waiting times at clinic [17,27,28]. Additionally, several studies pointed out that the distance to the health facilities is one of the major barriers that were raised by the majority of pregnant women in resource-limited settings, especially in rural, agricultural villages [8,15,18,19].

Despite the importance of distance to health facilities, only a few studies have accurately measured the actual travel distance between the villages where pregnant women live and the nearest health center where they can receive ANC. Most previous studies have used straight-line distances (Euclidean distances) as a proxy for actual travel distance [29] or relied on distance information obtained from respondents [18,19]. In rural areas with limited infrastructure and road systems, it is quite common that the actual travel distance is much longer than the straight-line distance. Using straight-line distances have been considered inaccurate, because it ignores geographical/environmental barriers such as rivers, mountains, road types, and road undulation [30,31]. Although straight-line distance can be measured easily on a map, relying on this data can result in grossly underestimating the burden put on pregnant women to access ANC. Additionally, respondents’ answers regarding distance to the nearest health facility can be unreliable and may vary even if they live in the same village. As lack of access has been established as a barrier to women receiving ANC, any intervention targeting these women needs to accurately take into account the actual travel distance and geographical/environmental obstacles they may have to face during their travels. To this end, we focused on accurately measuring the travel distance using a geographic information system (GIS) [31].

Cambodia is one of the Southeast Asian countries that has been suffering from low utilization of maternal and child health services as well as high maternal and child mortality. In 2015, Cambodia had the third highest rates for neonatal mortality (15 per 1,000 live births), under-five mortality (29 per 1,000 live births), and maternal mortality (161 per 100,000 live births) in Southeast Asia (regional average: 13 per 1,000, 27 per 1,000, and 110 per 100,000 live births, respectively) [32]. A recent study in Cambodia demonstrated that as many as 40% of Cambodian women did not receive all three MNCH services (namely ANC), delivery with skilled-birth attendance, and PNC in 2010 [11].

As observed in many developing countries, there is substantial regional variation in the use of maternal and child health services in Cambodia. In the Ratanakiri province, consisting mainly of rural, agricultural villages with the lowest socioeconomic standards and limited infrastructure in the country, the percentage of pregnant women and mothers who received three kinds of MNCH services was only 24% compared to 96% in Phnom Penh in 2010 [11]. Additionally, in the same Ratanakiri province, the Demographic Health Survey (DHS) conducted in 2014 found that less than half (48.4%) of the 76 pregnant women were reported to have received ANC four times or more, which percentage was much lower than that in Phnom Penh (88.0%) [33].

Despite the serious situation, few studies have examined the barriers for pregnant women to receiving ANC in rural Cambodia. Moreover, no previous studies have accurately measured actual travel distance and examined geographical/environmental obstacles for pregnant women to reach health facilities that provide ANC.

This study, therefore, aims to 1) describe the current situation of ANC use among pregnant women living in rural, agricultural villages in the Ratanakiri province, Cambodia, and to 2) examine barriers to accessing ANC, using data from a community-based cross-sectional survey and a GIS. Based on the results of this study, factors associated with ANC use, barriers to access ANC, and the importance of measuring distance accurately for developing strategies to increase ANC are discussed.

## Methods

### Study site

This study was conducted as a baseline survey for an intervention study, which is currently carried out to integrate community-based malaria control and MNCH services in the Ratanakiri province, Cambodia. Ratanakiri province is one of the least developed provinces, with low health standards and socioeconomic status, in the country [33]. It is located in the north-eastern part of the country and shares its borders with Lao People’s Democratic Republic (PDR) and Vietnam. Residents, including 13 ethnic minority groups, generally live in villages of 20–60 families and mainly engage in subsistence shifting agriculture, rice farming, and plantation of rubber, cashew, and cassava.

Multi-stage sampling was conducted to select study villages. First, 12 out of 27 health centers in Ratanakiri, which cover villages where village malaria workers (VMW) reside and work on malaria control for their villagers, were chosen (VMW villages). Out of the 12 health centers, seven were selected based on accessibility throughout the year: Andaung Meas, Chomrom Bay Sruk, Kachanh, Lumphat, Ochum, Oyadav, and Taveng. Out of 73 VMW villages under the seven health centers, ten villages under the Ochum health center were excluded due to difficulty of access during the rainy season, and one village under the Andaung Meas health center was excluded because of the absence of eligible interviewees at the time of the survey. As a result, 62 VMW villages were selected for the study.

### Data collection

#### Survey data

Data were collected from December 23 to 30, 2015, through interviews with mothers with children under two living in the selected VMW villages in the Ratanakiri province. To conduct face-to-face interviews, a semi-structured questionnaire was developed. The majority of the questions were drawn from three questionnaires: 1) Cambodia Demographic Health Survey 2014 [33], 2) questionnaires that we have already used in Cambodia for our previous studies on VMWs and mothers with children under five [34–36], and 3) a questionnaire that we used for the Ghana EMBRACE Implementation Research Project in collaboration with Ghana Health Services [7,37]. Specifically, the questionnaire contained questions regarding the following: a) mothers’ and their families’ socio-demographic characteristics; b) history of receiving MNCH services (frequency and timing of receiving ANC, delivery with SBA, PNC); c) history of malaria infection and testing during pregnancy (frequency, timing, through MNCH services or not); d) barriers to accessing health facilities; e) health-related behavior of mothers for themselves and their children under two (bednet use, breastfeeding, complementary feeding, treatment seeking behavior, etc.); f) mothers’ complications during pregnancy and childbirth (anemia, bleeding, etc.); g) children’s complications (fever, difficult breathing, etc.); and h) history of infectious diseases (malaria, diarrhea, and pneumonia, etc.). The questionnaire was first developed in English, translated into Khmer by Cambodian health experts, and then double-checked by other Khmer health experts to make sure that the translation was accurate.

Seventeen surveyors were hired to visit each survey participant’s residence and to conduct face-to-face interviews. Prior to the interview, experts from the National Center for Parasitology, Entomology, and Malaria Control, Cambodia (CNM) and lead researchers conducted a one-day training for the surveyors to explain the details of the questionnaire and how to conduct the interviews.

Surveyors were divided into four groups and visited 62 study villages within eight days. (Each survey team visited 14–17 villages in total.) In each study village, surveyors visited residences of mothers with children under two based on the information provided by VMWs. We recruited all mothers with children under two, who were available at the time of the survey. If mothers were absent, a second attempt was made within the same day. Those who were absent during the second visit and who were physically or mentally unable to provide informed consent were excluded from the study. There were no mothers who refused to participate in the study. A total of 388 mothers were interviewed. During the interview, surveyors asked mothers to show them maternal health record book and confirmed the accuracy of mothers’ report about the number and timing of ANC visits. Out of the 388 mothers interviewed, 11 were excluded from analysis because of missing data.

#### GPS data collection and analysis

Simultaneously with the survey, GPS data were collected to exactly measure actual travel distance between 62 villages and their nearest health center. Four GPS data collectors were hired and actually traveled from the center of each village to its nearest health center as taught by VMWs and/or study participants living in the village. Village center was designated as the starting point of the travel because households are clustered around the village center, where a meeting place or an open space with a well locates. Drivers traveled with with Holux m-241, a wireless GPS logger, which was set to record GPS data, including latitude, longitude, altitude, and time, every five seconds during each trip. Data were first entered into Excel 2016 through Holux exTour for Logger v2.1 software, and then into Arc Map 10.4, which was used to calculate actual travel distance between each village and its nearest health center. In addition, straight-line distance was also calculated using the same software, Arc Map 10.4, to compare with actual travel distance. Means of transportation and obstacles that mothers faced during their travel were asked in the face-to-face interview.

#### Data management, statistical analysis, and ethical considerations

All survey data were coded, entered into data analysis software with actual travel distance data and straight-line distance data, and then double-checked by the authors to ensure accuracy. Descriptive analysis was conducted to describe sociodemographic characteristics, ANC service use, knowledge of ANC, presence of complications during pregnancy, and perceived barriers to receiving ANC among mothers who attended ANC four times or more (ANC4+ achievers) and those who received ANC three times or less or not at all (non-achievers). Mothers were assigned to socio-economic status quartiles based on household assets and housing characteristics determined by principal component analysis [36,38].

To identify determinants of achievements in receiving ANC four times or more, two multiple logistic regression analyses were performed: one with actual travel distance and the other with straight-line distance. By doing so, we aimed to detect discrepancies or inconsistencies between the two distance measures, if any. In each analysis, the following independent variables were included: age, education, husband’s education, wealth quartiles, ethnicity, number of children, presence of health issues during pregnancy, experience of stillbirth and/or child loss, the nearest health center, and knowledge about ANC (if ANC is recommended even without any health issues, and recommended times of ANC). Out of the other sociodemographic characteristics, literacy was excluded from the regression analysis because of multicollinearity with education (r = 0.89). Marital status and occupation were excluded because of the small sub-group sample sizes (too few mothers who were not married and who were not farmers). Child age and child gender were also excluded because they are irrelevant to ANC use during the pregnancy.

A variety of perceived barriers to accessing health center among ANC4+ achievers and non-achievers are summarized with descriptive statistics. In the questionnaire, twenty-seven possible perceived barriers that mothers could have felt when accessing or trying to access ANC were asked with four choices: yes, to some extent, no, and don’t know. Possible perceived barriers asked in the questionnaire were selected based on previous studies: a financial barrier, a transportation barrier, seven geographical/environmental barriers, eight health-related barriers, six facility-related barriers, and five family-related barriers [5,15,39]. Those who answered “yes” and “to some extent” were classified as those who perceived the barrier. Open-ended questions were also asked if mothers felt any other barriers, but all answers overlapped with one of the given 27 barriers.

Data analysis was done using STATA version 14. Informed consent was obtained from all study participants before the interview. The study protocol, consent forms, and survey questionnaires were approved by the National Ethics Committee for Health Research, the Ministry of Health, Cambodia (368 NECHR), the Research Ethics Committee of Tokyo University of Agriculture and Technology (28–45), and the Research Ethics Committee of the University of Tokyo (11030).

## Results

A total of 377 mothers with children under two living in 62 villages in the Ratanakiri province, Cambodia were included in the analysis for this study. Their sociodemographic characteristics are described in Table 1. Approximately half (50.9%) of the mothers were in their twenties, and about a fourth (23.7%) were in their thirties. Most mothers were married (97.6%). Two thirds (66.6%) of the mothers had no school education at all. Two thirds (68.2%) responded that they were illiterate, and only 5.3% were confident about their ability to read. Their husbands were slightly better educated than their wives, but about 45% had no school education. Most of the mothers (91.3%) were farmers. In addition to mothers who belonged to the Khmer group, which is the largest ethnic group in Cambodia, there were ten other ethnic minority groups represented in this study. Of these, three groups comprised about two-thirds of the study population (Thompoun 23.4%, Charay 20.2%, and Kreung 15.4%).

**Table 1:** Sociodemographic characteristics of ANC4+ achievers and non-achievers (Total n = 377 in 62 villages).

Characteristics	Overall (n = 377)		Achievers (n = 123)		Non-achievers (n = 254)		
	n	%	n	%	n	%	p-value
Age							
<20	80	21.2	24	19.5	56	22.0	0.055
20–29	192	50.9	71	57.7	121	47.6	
30–39	89	23.7	27	22.0	62	24.4	
40+	16	4.2	1	0.8	15	5.9	
Marital status (Married)	368	97.6	122	99.2	246	96.9	0.540
Education							
None	251	66.6	59	48.0	192	75.6	**<0.001**
Primary (1–6 years)	98	26.0	46	37.4	52	20.5	
≧Secondary (≧7 years)	28	7.4	18	14.6	10	3.9	
Literacy							
Cannot read at all	257	68.2	65	52.8	192	75.6	**<0.001**
Able to read only part	100	26.5	45	36.6	55	21.7	
Able to read whole	20	5.3	13	10.6	7	2.8	
Husband’s education							
None	170	45.1	34	27.6	136	53.5	**<0.001**
Primary (1–6 years)	137	36.3	56	45.5	81	31.9	
≧Secondary (≧7 years)	70	18.6	33	26.8	37	14.6	
Couple’s education							
None	177	46.9	38	30.9	139	54.7	**<0.001**
≧Primary (≧1 year)	200	53.1	85	69.1	115	45.3	
Occupation (farmer)	344	91.3	108	87.8	236	92.9	0.100
Wealth quartiles							0.312
Low	95	25.2	26	21.1	69	27.2	
Lower middle	94	24.9	28	22.8	66	26.0	
Upper middle	94	24.9	32	26.0	62	24.4	
High	94	24.9	37	30.1	57	22.4	
Ethnicity							
Khmer	42	11.2	27	22.0	15	5.9	**<0.001**
Thompoun	88	23.4	25	20.3	63	24.8	
Charay	76	20.2	20	16.3	56	22.0	
Kreung	58	15.4	19	15.4	39	15.4	
Other	113	29.8	32	26.0	81	31.9	
Number of children							
1	140	37.2	45	36.6	95	37.4	0.152
2	112	29.7	45	36.6	67	26.4	
3–4	99	26.2	27	22.0	72	28.3	
5+	26	6.9	6	4.9	20	7.9	
Child age (month)							
0–5	141	37.4	96	78.0	45	17.7	0.190
6–11	96	25.5	59	48.0	37	14.6	
12–17	73	19.3	56	45.5	17	6.7	
18–23	67	17.8	43	35.0	24	9.4	
Sex of the child (female)	181	48.0	64	52.0	117	46.1	0.274
Experience of stillbirth (Yes)	42	11.1	14	11.4	28	11.0	0.917
Experience of child loss* (Yes)	49	13.0	16	13.0	33	13.0	0.997
Health center							
Oyadav (17 villages)	93	24.7	18	14.6	75	29.5	**0.006**
Lumphat (8)	75	19.9	31	25.2	44	17.3	
Andaung Meas (10)	64	17.0	27	22.0	37	14.6	
Ochum (10)	50	13.2	15	12.2	35	13.8	
Taveng (8)	38	10.1	16	13.0	22	8.7	
Chomrom Bey Sruk (3)	29	7.7	5	4.1	24	9.4	
Kachanh (6)	28	7.4	11	8.9	17	6.7	
Actual travel distance to health center (km)							
< 5.0	42	11.1	18	14.6	24	9.4	**0.001**
5.0–9.9	98	26.0	44	35.8	54	21.3	
10.0–14.9	132	35.0	41	33.3	91	35.8	
15.0 +	105	27.9	20	16.3	85	33.5	
Straight-line distance to health center (km)							
< 5.0	67	17.8	33	26.8	34	13.4	**0.003**
5.0–9.9	162	43.0	53	43.1	109	42.9	
10.0–14.9	75	19.9	22	17.9	53	20.9	
15.0 +	73	19.3	15	12.2	58	22.8	

* Experience of child loss: Mother had lost a child/children after delivery.

About a third (37.2%) of the mothers had one child. Child age (months) varied, but about a third (37.4%) belonged to the youngest age group, 0–5 months. About half (48.0%) of the children were female. Approximately a tenth (11.1%) of the mothers had experienced stillbirth, and 13.0% had lost children in the past.

**Fig 1:**
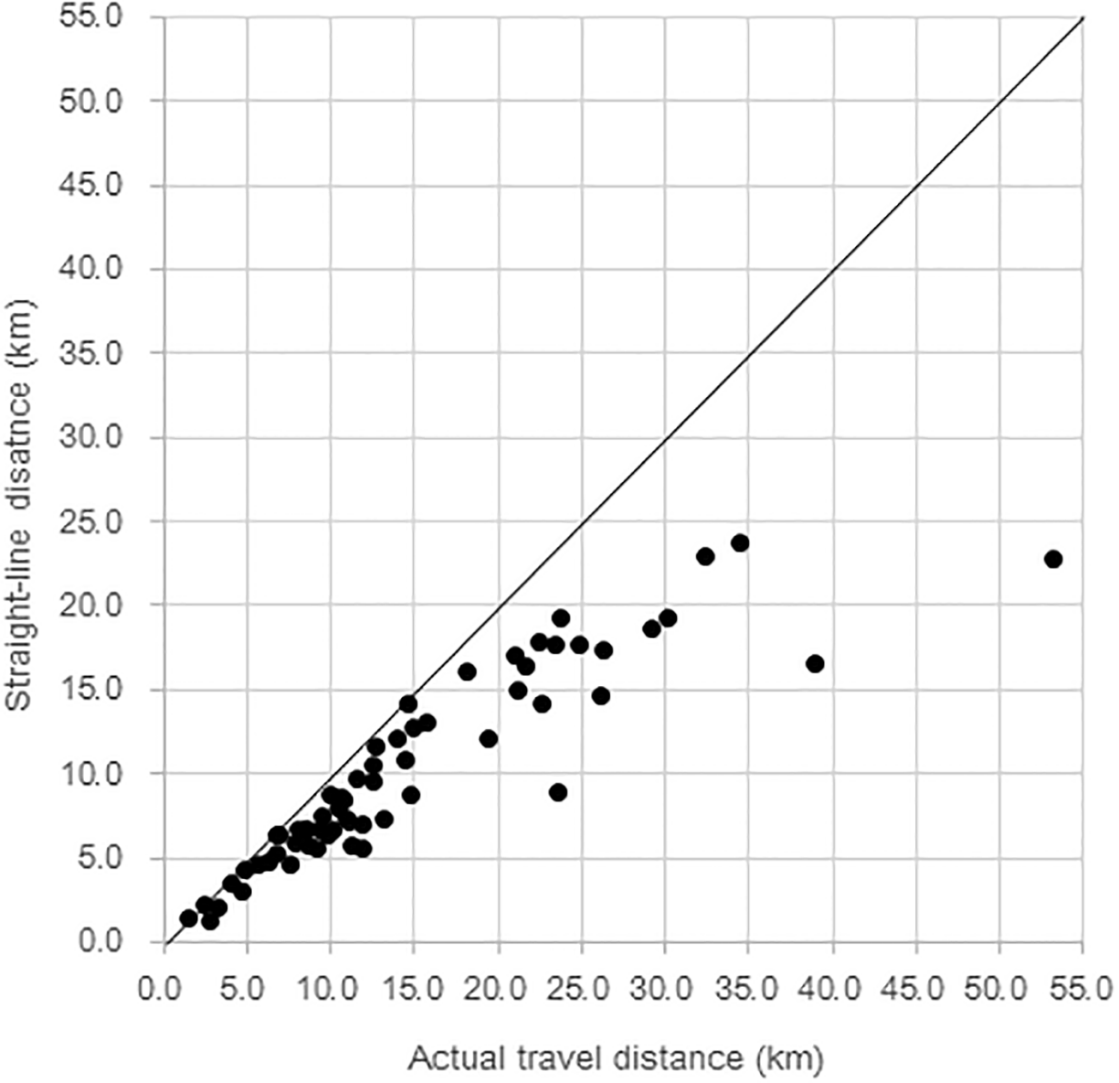
Discrepancy between actual travel distance and straight-line distance from 62 villages to their nearest health centers in Ratanakiri province.

**Fig 2:**
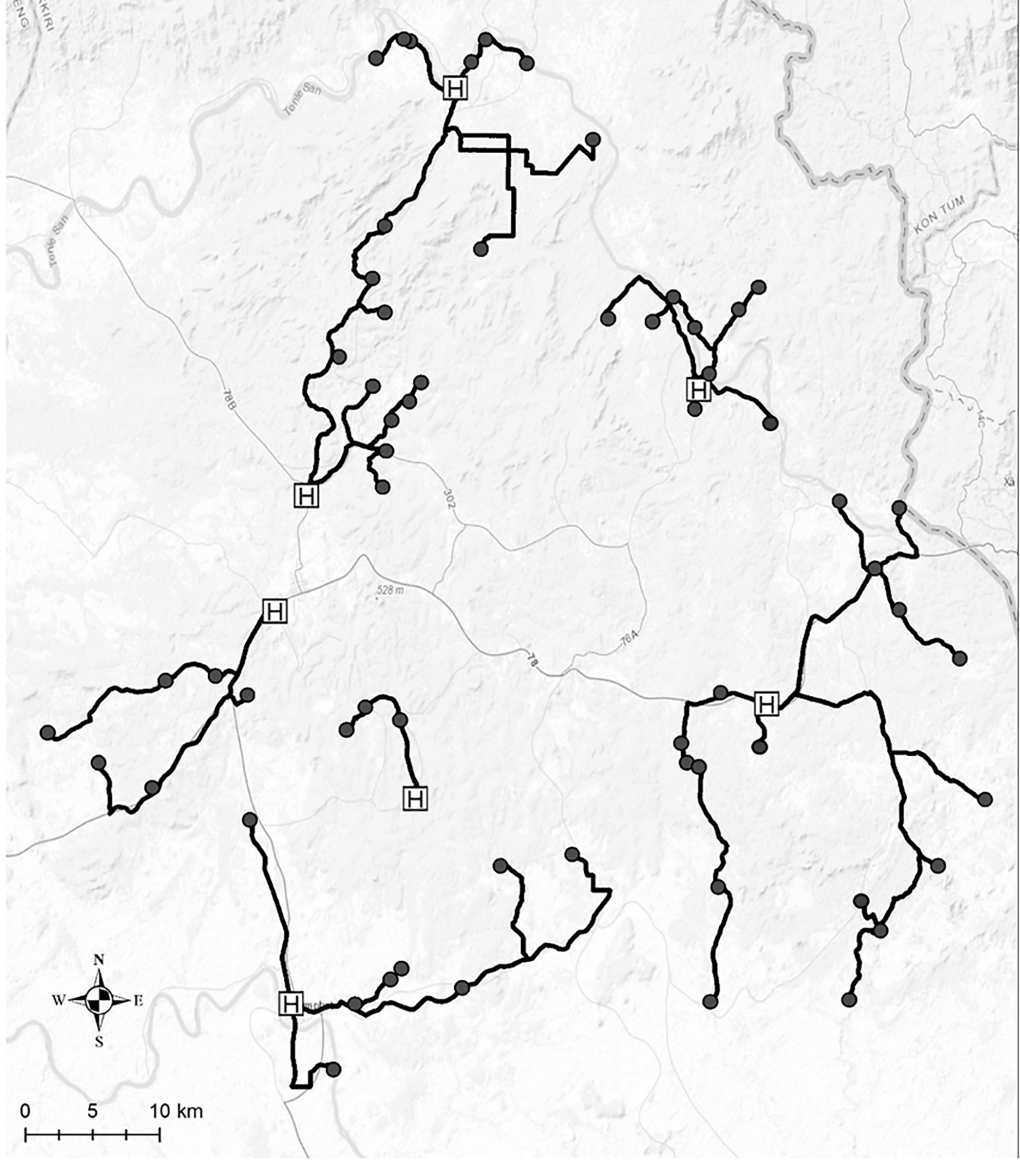
Map of study site in Ratanakiri province with actual travel route from 62 villages to their nearest health centers. Black circle: village, black line: actual travel route from each village to its nearest health center, H: health center (Sources: Esri, HERE, DeLorme, Intermap, increment P Corp., GEBCO USGS, FAO, NPS, NRCAN, GeoBase, IGN, Kadaster NL, Ordnance Survey, Esri Japan, METI Esri China (Hong Kong), swisstopo, MapmyIndia, OpenStreetMap contributors, and the GIS User Community).

Actual travel distance between each of the 62 villages and the nearest health center was measured using the GIS and compared with straight-line distance measured on a map. A striking discrepancy was found between data gained by the two distance measures (Fig 1). Depending on the village, difference between actual travel distance and straight-line distance ranged from 3.0 m to 30.2 km. For all villages, actual travel distance measured using GPS was longer than the straight-line distance (median difference = 2.83km, mean difference = 4.48 km, SD = 5.19). The mean of the actual travel distance between 62 villages and their nearest health centers (14.71 km, SD = 9.84) was significantly longer than the mean of the straight-line distance (10.23 km, SD = 5.75) (t = 6.79, df = 61, p<0.001). As shown in Fig 2, pregnant women living in the majority of villages had to make a detour from the straight-line path because of insufficient road networks and environmental barriers such as forests and rivers.

The distances the mothers had to travel are distributed very differently between actual and straight-line distances (chi-square test p<0.001) (Table 1). According to the actual travel distance, 11.1% of the mothers were found to have lived in villages where the distance to the nearest health center was shorter than 5.0 km. About a fourth (26.0%) lived in villages with a distance between 5.0 km and 9.9 km, 35.0% between 10.0 km and 14.9 km, and 27.9% belonged to the farthest group of 15 km or longer. However, based on the straight-line distance, nearly half (43%) of the mothers were found to have lived in villages 5.0–9.9 km away, and only 19.9% and 19.3% were found to have lived in villages of 10.0–14.9 km away and 15.0 km or farther from the nearest health center, respectively.

Similarly, the two distance measures showed totally different distributions of ANC4+ and non-achievers. Actual travel distance showed that the majority (71.3%) of the non-achievers lived in villages 10.0 km or farther from the nearest health center. On the other hand, the straight-line distance showed less than half (43.7%) lived in villages 10.0 km or farther, while nearly the other half (42.9%) lived in villages 5.0–9.9 km away from the nearest health center. These findings clearly show that straight-line distances are misleading because they convey the impression that the study population as a whole and ANC non-achievers live closer to the nearest health center than in reality.

Frequency of ANC use, knowledge of ANC, contents of health education received at ANC, and experience of health issues during pregnancy are summarized in Table 2. Only about a third (32.6%) of the mothers had received ANC four times or more as recommended by the WHO [40]. About a quarter (24.1%) of the mothers never received ANC during their most recent pregnancy. Most (92.7%) of the mothers, who had been to the nearest health center to receive ANC, took a motorcycle, which was owned by their family or someone in their village. The majority of mothers (71.9%) were aware that receiving ANC is recommended regardless of the presence of health problems. However, only about a third (31.3%) of them knew that pregnant women are recommended to receive ANC at least four times. Only 13.5% of the mothers were aware of one or more of the danger signs during pregnancy such as severe headache, vaginal bleeding, and vaginal secretion. Most mothers reported that they received ANC because they were willing to get a checkup (55.9%) or they were worried about their pregnancy (25.5%) or health (9.8%).

**Table 2:** ANC utilization and knowledge among ANC4+ achievers and non-achievers.

	Overall		Achievers		Non-achievers		
	n	%	n	%	n	%	p-value
ANC visit (times)	(n = 377)		(n = 123)		(n = 254)		
0	91	24.1	0	0	91	35.8	
1	41	10.9	0	0	41	16.1	
2	48	12.7	0	0	48	18.9	
3	74	19.6	0	0	74	29.1	
4 or more	123	32.6	123	100	0	0	
Transportation to ANC (n = 286)	(n = 286)		(n = 123)		(n = 163)		
Motorcycle	265	92.7	114	92.7	151	92.6	0.340
Taxi	4	1.4	1	0.8	3	1.8	
On foot	3	1.1	0	0.0	3	1.8	
Other (bicycle, boat etc.)	14	4.9	8	6.5	6	3.7	
Knowledge							
ANC is recommended regardless of the presence of complications	271	71.9	111	90.2	160	63.0	**<0.001**
Recommended times of ANC (4 or more)	118	31.3	68	55.3	50	19.7	**<0.001**
Danger sign during pregnancy (correct)	51	13.5	28	22.8	23	9.1	**<0.001**
Major reason why received ANC	(n = 286)		(n = 123)		(n = 163)		
Wanted to get a checkup	160	55.9	71	57.7	89	54.6	0.419
Worried about pregnancy	73	25.5	32	26.0	41	25.2	
Worried about health problem	28	9.8	11	8.9	17	10.4	
Health workers’ advice	1	0.3	0	0.0	1	0.6	
Family’s suggestion	5	1.7	0	0.0	5	3.1	
Other	19	6.6	9	7.3	10	6.1	
Knowledge gained at ANC (Multiple choice)	(n = 286)		(n = 123)		(n = 163)		
Recommended place of delivery	222	77.6	104	84.6	118	72.4	**0.015**
Recommended timing and frequency of ANC	167	58.4	77	62.6	90	55.2	0.210
Early initiation and exclusively breastfeeding	142	49.7	65	52.8	77	47.2	0.348
Danger signs during pregnancy	138	48.3	63	51.2	75	46.0	0.383
Nutrition of pregnant women	132	46.2	61	49.6	71	43.6	0.311
Items to prepare before delivery	125	43.7	65	52.8	60	36.8	**0.007**
Family planning	109	38.1	61	49.6	48	29.4	**0.001**
How to prevent malaria	84	29.4	39	31.7	45	27.6	0.451
Arrange transportation to go to HC for delivery	62	21.7	34	27.6	28	17.2	**0.033**
Presence of health issues during pregnancy	(n = 377)		(n = 123)		(n = 254)		
	126	33.4	43	35.0	83	32.7	0.660
Kinds of health issues during pregnancy (Multiple choice)	(n = 377)		(n = 123)		(n = 254)		
severe headache	88	23.3	30	24.4	58	22.8	0.738
Fits	79	21.0	27	22.0	52	20.5	0.741
Fever	73	19.4	26	21.1	47	18.5	0.544
severe abdominal pain	60	15.9	18	14.6	42	16.5	0.636
fast/difficult breathing	27	7.2	10	8.1	17	6.7	0.612
vaginal bleeding	4	1.1	2	1.6	2	0.8	0.598
Received care to treat health issues	(n = 126)		(n = 43)		(n = 83)		
	74	58.7	29	67.4	45	54.2	0.134

Overall, higher percentages of achievers correctly answered each and every knowledge item compared to non-achievers. Significantly higher percentages of achievers learned about the recommended place of delivery (84.6% vs 72.4%, p = 0.015), items to prepare before delivery (52.8% vs 36.8%, p = 0.007), family planning (49.6% vs 29.4%, p = 0.001), and the necessity of arranging transportation to go to HC for delivery (27.6% vs 17.2%, p = 0.033).

About a third (33.4%) of the mothers reported that they had experienced one or more health issues (complications) during pregnancy. The complications included severe headache (23.3%), fits (21.0%), fever (19.4%), severe abdominal pain (15.9%), fast/difficult breathing (7.2%), and vaginal bleeding (1.1%). A higher percentage of mothers who achieved ANC4+ received care for the complications compared to non-achievers, but the difference was not statistically significant (67.4% vs 54.2%, p = 0.134).

Factors associated with achieving ANC4+ were examined by multiple logistic regression analysis (Table 3). Two models were run, one with actual distance data and the other with straight-line distance data. In both models, significant promoting factors for achieving ANC4+ were mother’s educational background and knowledge about ANC, while the only preventing factor was distance to the nearest health center where pregnant women can receive ANC. According to the model using actual travel distance, mothers with secondary or higher education were about five times more likely to have achieved ANC4+ during their most recent pregnancy (AOR = 5.50, 95%CI: 1.74, 17.37). Mothers who were aware that ANC is recommended for pregnant women even without any health issues were about three times more likely to have achieved ANC4+ (AOR = 2.74, 95%CI: 1.25, 6.00), and those who knew the recommended times for ANC were about four times more likely to have achieved ANC4+ (AOR = 2.26, 95%CI: 7.22). Mothers who had to travel 10.0–14.9km to get to the nearest health center were about 70% less likely to have achieved ANC4+ (AOR = 0.32, 95%CI: 0.10, 0.99), and those who had to travel 15.0 km or longer were about 80% less likely to have achieved ANC 4+ (AOR = 0.21, 95%CI: 0.07, 0.62) compared to those who lived in villages with actual travel distance of 5.0 km or less.

**Table 3:** Factors associated with achieving ANC4+ (n = 377).

	Univariate			Multiple logistic regression with actual travel distance			Multiple logistic regression with straight-line distance		
	OR	95%CI		AOR	95%CI		AOR	95%CI	
Age									
<20	1			1			1		
20–29	1.37	0.78	2.40	1.00	0.46	2.22	0.98	0.45	2.15
30–39	0.85	0.45	1.62	0.73	0.26	2.00	0.76	0.28	2.06
Education									
None	1			1			1		
Primary (1–6 years)	**2.88**	1.76	4.71**	1.70	0.84	3.41	1.73	0.86	3.50
≧Secondary (≧7 years)	**5.86**	2.56	13.38**	**5.50**	1.74	17.37**	**5.08**	1.63	15.82*
Husband’s education									
None	1			1			1		
Primary (1–6 years)	2.77	1.67	4.59	1.69	0.86	3.32	1.64	0.83	3.24
≧Secondary (≧7 years)	3.57	1.96	6.51	1.58	0.66	3.75	1.47	0.61	3.53
Wealth quartiles									
Low	1			1			1		
Lower middle	1.13	0.60	2.12	1.08	0.49	2.35	1.01	0.47	2.18
Upper middle	1.37	0.74	2.55	1.73	0.80	3.76	1.70	0.79	3.68
High	1.72	0.93	3.18	1.28	0.57	2.90	1.26	0.57	2.82
Ethnicity									
Khmer	1			1			1		
Thompoun	**0.22**	0.10	0.48**	0.52	0.16	1.72	0.60	0.19	1.94
Charay	**0.20**	0.08	0.45**	0.50	0.13	1.94	0.47	0.13	1.80
Kreung	**0.27**	0.12	0.62**	1.07	0.30	3.80	1.17	0.32	4.25
Other	**0.22**	0.10	0.47**	0.39	0.13	1.20	0.38	0.12	1.16
Number of children									
1	1			1			1		
2	1.42	0.85	2.38	2.05	0.97	4.35	2.02	0.96	4.27
3–4	0.79	0.45	1.40	0.96	0.38	2.42	0.91	0.37	2.28
5+	0.63	0.24	1.69	1.20	0.31	4.74	1.13	0.29	4.44
Presence of danger sign during pregnancy									
Not present	1			1			1		
Present	1.11	0.70	1.74	1.17	0.66	2.10	1.22	0.68	2.18
Experience of stillbirth and/or child loss									
No	1			1			1		
Yes	0.87	0.48	1.58	1.14	0.52	2.53	1.06	0.48	2.33
Health center									
Oyadav	1			1			1		
Lumphat	**2.94**	1.47	5.85**	2.21	0.59	8.23	1.84	0.53	6.32
Andaung Meas	**3.04**	1.49	6.21**	1.94	0.74	5.11	2.13	0.82	5.57
Ochum	1.79	0.81	3.95	0.73	0.17	3.10	0.72	0.18	2.89
Taveng	**3.03**	1.33	6.91**	2.27	0.68	7.54	2.48	0.77	8.00
Chomrom Bey Sruk	0.87	0.29	2.59	0.54	0.11	2.78	0.48	0.98	2.38
Kachanh	**2.67**	1.08	6.74*	1.16	0.28	4.77	1.13	0.27	4.80
Actual travel distance to health center (km)									
< 5.0	1			1					
5.0–9.9	1.09	0.52	2.25	0.66	0.22	1.97			
10.0–14.9	0.60	0.29	1.23	**0.32**	0.10	0.99*			
15.0 +	**0.31**	0.14	0.69**	**0.21**	0.07	0.62**			
Straight-line distance to health center (km)									
< 5.0	1						1		
5.0–9.9	**0.50**	0.28	0.90*				0.46	0.19	1.08
10.0–14.9	**0.43**	0.21	0.85*				**0.28**	0.10	0.79*
15.0 +	**0.27**	0.13	0.56**				**0.23**	0.08	0.64**
Knowledge: ANC is recommended									
No	1			1			1		
Yes	**5.43**	2.84	10.34**	**2.74**	1.25	6.00*	**2.82**	1.28	6.23*
Knowledge: Recommended times of ANC									
Wrong	1			1			1		
Correct (four or more)	**5.04**	3.15	8.08**	**4.04**	2.26	7.22**	**4.07**	2.28	7.29**

*p<0.05

**p<0.01

Details of perceived barriers that mothers felt in accessing the nearest health center to receive ANC during their most recent pregnancy were compared between ANC 4+ achievers and non-achievers (Table 4). A significantly higher percentage of non-achievers perceived financial barriers compared to achievers (77.6% vs 56.9%, p<0.001). In addition, distance and undulation of the road to the nearest health center were geographical/environmental barriers that were perceived by a significantly higher percentage of non-achievers compared to achievers (85.4% vs 77.2%, p = 0.048; 70.1% vs 58.5%, p = 0.026). Abdominal pain, being unaware of where to receive ANC, and having no one to accompany one were also important barriers that were perceived by a significantly higher percentage of non-achievers compared to achievers (46.5% vs 35.8%, p = 0.049; 13.0% vs 4.9%, p = 0.015; 25.2% vs 9.8%, p<0.001). All raw research data and codes for variables are shown in S1, S2 and S3 Files.

**Table 4:** Perceived barriers to access health center among ANC4+ achievers and non-achievers.

Perceived barrier	Overall (n = 377)		Achievers (n = 123)		Non-achievers (n = 254)		
	n	%	n	%	n	%	p-value
Financial barrier							
No money	300	79.6	91	74.0	209	82.3	0.061
Transportation barrier							
No transportation	267	70.8	70	56.9	197	77.6	**<0.001**
Geographical/Environmental barrier							
Too far	312	82.8	95	77.2	217	85.4	**0.048**
Bumpy road	267	70.8	81	65.9	186	73.2	0.140
Dust	266	70.6	85	69.1	181	71.3	0.667
Undulation of the road	250	66.3	72	58.5	178	70.1	**0.026**
Rain	216	57.3	66	53.7	150	59.1	0.321
Forest/bush	89	23.6	25	20.4	64	25.2	0.296
Flood	63	16.7	22	17.5	41	16.1	0.670
Health-related barrier							
Fetal movement	248	65.8	76	61.8	172	67.7	0.255
Felt tired	245	65.0	75	61.0	170	66.9	0.256
Headache	218	57.8	64	52.0	154	60.6	0.279
Vomiting	203	53.8	66	53.7	137	53.9	0.959
Abdominal pain	162	43.0	44	35.8	118	46.5	**0.049**
Blurry vision	122	32.4	37	30.1	85	33.5	0.510
Edema	94	24.9	24	19.5	70	27.6	0.090
Vaginal bleeding	13	3.4	4	3.3	9	3.5	0.574
Facility-related barrier							
Long waiting time	175	46.4	64	52.0	111	43.7	0.128
Inconvenient service hour	131	34.7	48	39.0	83	32.7	0.225
Not wanting to be scolded	91	24.1	27	22.0	64	25.2	0.490
Afraid to go	90	23.9	19	15.4	71	28.0	**0.008**
Good service not available	46	12.2	10	8.1	36	14.2	0.093
Didn’t know where to go	39	10.3	6	4.9	33	13.0	**0.015**
No female doctor	29	7.7	5	4.1	24	9.4	0.066
Family-related barrier							
Difficult to leave elder children (achiever = 78, non-achiever = 159)	77	32.5	23	29.5	54	34.0	0.489
No one can accompany	76	20.2	12	9.8	64	25.2	**<0.001**
No support from husband	57	15.1	14	11.4	43	16.9	0.159
No support from mother-in-law	39	10.3	13	10.6	26	10.2	0.921

## Discussion

This study revealed the limited use of ANC and barriers for pregnant women living in rural, agricultural villages to accessing ANC in the Ratanakiri province, Cambodia. Only a third of the mothers had received ANC four times or more and a quarter of the mothers had never received ANC during their most recent pregnancy. Factors associated with achieving ANC4+ were mother’s educational background, knowledge about ANC, and actual travel distance to the nearest health facility. As for perceived barriers, compared to ANC4+ achievers, a significantly higher percentage of non-achievers perceived to have financial barriers, geographical/environmental barriers (actual travel distance and undulation of the road), as well as health-related (abdominal pain), facility-related (don’t know where to go), and family-related (no one could accompany the mother) barriers. Furthermore, the mothers’ lack of knowledge was quite evident. Although about a third of the mothers had experienced complications during their pregnancy, only one out of seven were aware of the danger signs during pregnancy.

ANC use was limited with only a third (32.6%) of the mothers having received ANC four times or more during their most recent pregnancy. This is much lower than the figure (48.4%) obtained by the DHS in Cambodia conducted in 2014 [33], as well as those reported by recent studies conducted in other developing countries, such as 86% on average from three locations in Ghana [41] and 46% in Rwanda [16]. Together with the fact that a fourth of the pregnant women had never received ANC, this study highlighted the urgent need to increase ANC among this population.

Despite the large discrepancy in reported ANC use, many of our results were consistent with other similar studies, assuring the validity of our findings. For example, mothers’ higher educational background and better knowledge about ANC were promoting factors for receiving ANC four times or more. Several previous studies have demonstrated that women’s education was positively associated with their health service use [8,18,39]. In addition, our study revealed that knowledge of recommended frequency of ANC is crucial for achieving ANC4+. Even though the majority of the mothers knew that receiving ANC is recommended, only a third of them were aware of the recommended frequency. Those who had this knowledge were four-times more likely to have achieved ANC4+. To effectively increase ANC use, this information needs to reach pregnant women ideally at the first ANC.

Also, similar to previous studies, we found that travel distance is negatively associated with achieving ANC4+. Previous studies have pointed out that distance could be a significant barrier for health service use. A qualitative study conducted in another rural province, Kampong Cham, in Cambodia demonstrated that distance to the health facility, poor road conditions, and inadequate transportation were common obstacles raised by reproductive age women to use health services [15]. Karra et al. reported that even relatively small distances from health facilities were associated with child mortality, and that reducing travel distances and travel times is likely to increase the use of health services and reduce neonatal mortality [42]. The negative effect of distance on health service use needs to be considered to develop an effective strategy to increase ANC and other maternal and child health service use.

Although travel distance has been established by others and confirmed in this study as a barrier to health service use, this study additionally elucidates the importance of measuring actual travel distance accurately in remote areas with limited road networks. Many previous studies that included distance as a factor for health service utilization, only used straight-line distance as a proxy for actual travel distance [29]. However, straight-line distance gotten simply by connecting two points on a map is often inaccurate as it ignores geographical/environmental barriers that might affect actual travel distance [30]. This is especially true in many of the developing countries where the pregnant women must make numerous detours to avoid any number of geographical/environmental barriers, such as forests, rivers, undulation of roads, etc. The importance of distinguishing between actual and straight-line distance was confirmed by our findings, as we found a statistically significant difference in distribution between straight-line distance and actual travel distance among ANC4+ achievers and non-achievers. Obviously, such a finding does not alter the known association between travel distance and ANC use, as evident by the results of the two multiple logistic regression models (with straight-line distance or actual distance) consistently showing that living in villages 10.0 km or farther from the nearest health center being negatively associated with achieving ANC4+. However, using actual travel distance, we found that a great majority of the ANC4+ non-achievers lived in villages that require trips of 10.0 km or longer to get to the nearest health center. Specifically, actual travel distance showed that more than two thirds of the ANC4+ non-achievers lived in villages 10.0 km or farther away, while straight-line distance showed that less than half of the non-achievers lived 10.0 km or farther away. Therefore, relying on straight-line distance creates the risk of misunderstanding the target population most in need of future intervention programs to assist in accessing ANC. Further, using actual distance may better account for the amount of effort and time it takes for a person to reach the nearest health facility, with the rationale that the longer actual distance is indicative of the fact that detours are being taken to avoid the many barriers that the person may encounter. Measuring actual travel distance using GPS logger is not time consuming (only one-way travel from a village to its nearest health center) and does not require access to governmental records or expensive equipment/facility. Data collection and analysis only require a handheld GPS and common software packages to accomplish accurate location of each travel path [43]. For future research that aims to analyze access to health services, especially in remote areas with limited road networks and geographical/environmental barriers, a GIS is recommended as a simple and cost-effective tool to measure distance accurately.

In addition to distance, other barriers to access ANC need to be considered when developing strategies or local health policies to raise health service use. Compared to ANC4+ achievers, a significantly higher percentage of non-achievers claimed undulation of the road, financial constraints, abdominal pain, being unaware of where to receive ANC, and having no one to accompany them as barriers when trying to access ANC. While most of these barriers have been described in several previous studies [5,15,17], undulation of the road has never been focused on as a barrier for pregnant women to access ANC. In addition to long-distance travel by motorcycle, undulation of the road can put pregnant women at risk of road accident, which can result in adverse fetal outcomes [44,45]. Future intervention programs and strategies to raise ANC use by pregnant women need to incorporate safety measures for travel. In addition, to tackle the above-mentioned barriers as a whole, it is highly recommended that community-based maternal health services and outreach programs tailored to the local context be conducted, especially in remote and resource-limited settings.

Findings from this study should be considered in the context of some limitations. First, mothers with children under two were asked about events during their most recent pregnancy, which might have introduced recall bias. Second, because interview data collected in this study were self-reported by mothers, there was a possibility of courtesy bias. To minimize these biases, data were confirmed with a maternal health record book whenever available, and interviews were conducted by experienced and trained interviewers with on-site supervision.

Despite these limitations, this is a pioneering study to examine barriers to accessing ANC, combining a community-based cross-sectional survey and a GIS. This study revealed factors associated with ANC use by pregnant women living in remote, agricultural villages in Cambodia. Furthermore, this study highlights the importance of measuring actual travel distance accurately to examine barriers to access health facilities and to properly select target populations for future interventions. The findings and methodology of this study will contribute to the development and implementation of an effective community-based intervention program to increase ANC use in Cambodia as well as in other developing countries, especially in remote areas with limited road networks.

## Supporting information

S1 File Raw research data. (XLSX)

S2 File Raw distance data. (XLSX)

S3 File Codes for variables. (XLSX)
